# Supplementary material for: Tumor site-directed A1R expression enhances CAR T cell function and improves efficacy against solid tumors
Source: Nat Commun. 2025 Jul 3;16:6123. doi: 10.1038/s41467-025-59021-9 (PMC12229354; doi:10.1038/s41467-025-59021-9)
Supplement: Supplementary file 1 — Supplementary Information [file 41467_2025_59021_MOESM1_ESM.pdf]

Supp Table 1: MYC-tagged hA1R peptide sequence

MEQKLISEEDLGGGGSPPSISAFQAAYIGIEVLIALVSVPGNVLVIWAVKVNQALRDATFCFIVSLAVADVAVGALVIPLAILINIGPQTYFHTCLM
NCITLFCPSCHKPSILTYIAIFLTHGNSAMNPIVYAFRIQKFRVTFLKIWNDHFRCQPAPPIDEDLPEERPDD*

Blue= G4S linker, Red= MYC tag

Supp Table 2: A1R Gene signature

A1R Signature UP	A1R Signature Down
ADAM19	AIF1
ADORA1	ANO9
AGPAT9	CAMK4
ALOX5AP	CCR7
ANP32E	CD5
ATP1B3	CD79A
BATF	CIRBP
BCAT1	COX7C
BIRC3	CYFIP2
CCL3	EEF1A1
CCL4	EEF2
CCR1	FAM102A
CD276	FAM134B
CD300C	FAM211A-AS1
CD40LG	FAM65B
CDC6	FAU
CDKN1A	GIMAP1
CEBPB	GRAMD1A
CHST2	H1FX
CKLF	HLA-A
CLECL1	IGFBP2
CLSPN	IL7R
COL6A3	KIT
CSF1	KRT86
CST7	LTB
DUSP2	MAL
DUSP5	MIR142
EGR1	MZT2A
EMP1	NACA
EPB41L2	NLRC5
G0S2	PCED1B-AS1
GNLY	PDCD4
GPR56	PIK3IP1
GRAMD1B	PLAC8
GZMA	RAB37
GZMB	RARRES3
GZMH	RPL10A
HBEGF	RPL11
HMGB1P17	RPL13
IER3	RPL13A
IFITM10	RPL14
IFNG	RPL18
IL13	RPL19
IL2RA	RPL3
JUNB	RPL30
KLRC1	RPL31

KLRD1	RPL32
LAG3	RPL34
MB	RPL35A
MBOAT7	RPL36
METRNL	RPL37
MT-ATP8	RPL37A
MT-CO1	RPL4
MT-ND1	RPL41
MT-ND5	RPL5
MT-ND6	RPL7A
MTHFD2	RPLP2
MYO1E	RPS12
NKG7	RPS14
PFKP	RPS15
PHLDA1	RPS18
PLAUR	RPS19
POLE3	RPS23
PPFIBP1	RPS24
PRRC2C	RPS27A
PTGS2	RPS28
PTMA	RPS3
RAB27A	RPS3A
RHOB	RPS4X
SETBP1	RPS5
SFXN1	RPS6
SH2D2A	RPS8
SLC27A2	SEC31B
SLC2A3	SELL
SRGN	38961
TBX21	SLC14A1
TFPI	SORL1
TIGIT	SPOCK2
TNF	SPPL2B
TOPBP1	STAT6
TPM4	SUN2
TUBA4A	TCF7
TUBB6	TNFRSF25
UBE2S	TRABD
ZBED2	TSPAN32
ZBTB32	TXNIP
ZFP36L1	UBA52
ZNF282	UBXN6
	YPEL3
	ZDHHC14
	ZSCAN16-AS1

Supp Fig 1

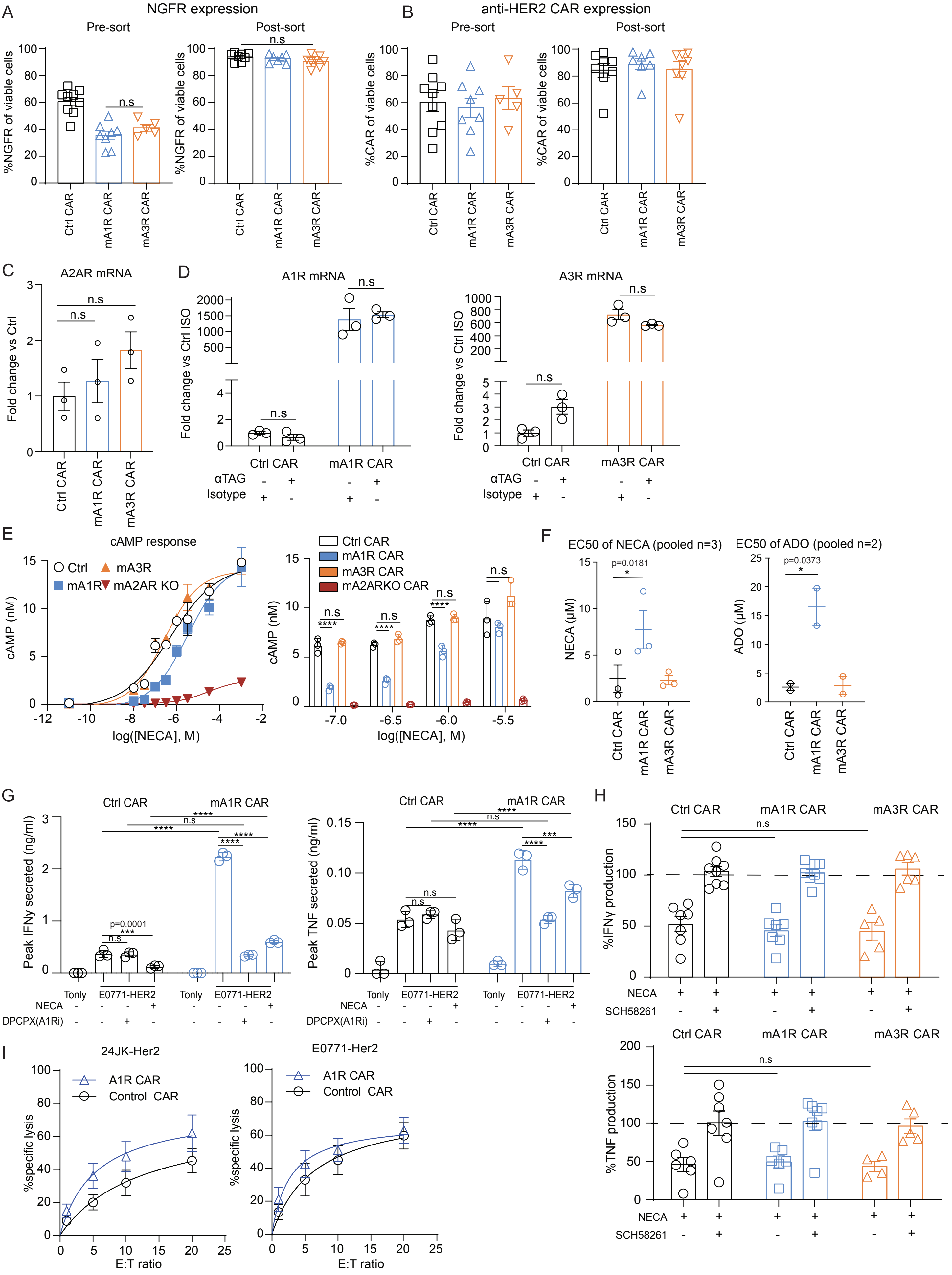
A
NGFR expression
Pre-sort
Post-sort
%NGFR of viable cells
n.s
Ctrl CAR
mA1R CAR
mA3R CAR
B
anti-HER2 CAR expression
Pre-sort
Post-sort
%CAR of viable cells
C
A2AR mRNA
Fold change vs Ctrl
n.s
D
A1R mRNA
A3R mRNA
Fold change vs Ctrl ISO
αTAG
Isotype
E
cAMP response
Ctrl
mA3R
mA1R
mA2AR KO
cAMP (nM)
log([NECA], M)
Ctrl CAR
mA1R CAR
mA3R CAR
mA2ARKO CAR
F
EC50 of NECA (pooled n=3)
EC50 of ADO (pooled n=2)
p=0.0181
p=0.0373
NECA (μM)
ADO (μM)
G
Ctrl CAR
mA1R CAR
Peak IFNγ secreted (ng/ml)
Peak TNF secreted (ng/ml)
p=0.0001
Tonly
E0771-HER2
NECA
DPCPX(A1Ri)
H
%IFNγ production
%TNF production
NECA
SCH58261
I
24JK-Her2
E0771-Her2
A1R CAR
Control CAR
%specific lysis
E:T ratio

Supplementary Figure 1: A_1R expression in mouse anti-HER2 CAR T cells. (A) %NGFR and (B) % anti-HER2 CAR positive T cells in pre- and post- NGFR bead sorted mouse CAR T cells from up to 9 individual experiments. (C-D) qRT-PCR to determine fold-expression versus control group of (C) $A_{2A}R$ and (D) A_1R or A_3R mRNA in stimulated/ unstimulated CAR T cells. Data represented as the mean ± SD of triplicate samples. (E) cAMP response curve and bar-plot quantification of control, mA_1R, mA_3R and $mA_{2A}R$ KO CAR T cells ($A_{2A}R_{-/-}$ mouse background) in response to increasing concentrations of pan-adenosine receptor agonist (NECA). Data represented as the mean ± SD of triplicate samples from a representative experiment of n = 2. (F) EC_{50} of NECA and eADO. (G-H) Coculture of murine anti-Her2 CAR T cells with E0771-Her2 tumor cells. (G) Cytokines secreted after two rounds of serial stimulation in the absence and presence of NECA (1µM) or DPCPX (100nM). Data represented as the mean ± SD of triplicate cultures. (H) Normalized cytokine secretion against no drug treatment for each group in the absence or presence of NECA (1µM) and SCH58261 ($A_{2A}R$ antagonist, 1µM) from up to 9 individual experiments. (I) Chromium release assay quantification of anti-Her2 CAR T cells cocultured with 24JK-HER2 sarcoma or E0771-HER2 tumor cells at indicated effector:target ratios. ****$p<0.0001$, ***$p<0.001$, **$p<0.01$, *$p<0.05$. (e, g, h, i) two-way ANOVA or (a, b, c, f) one-way ANOVA.

Supp Fig 2

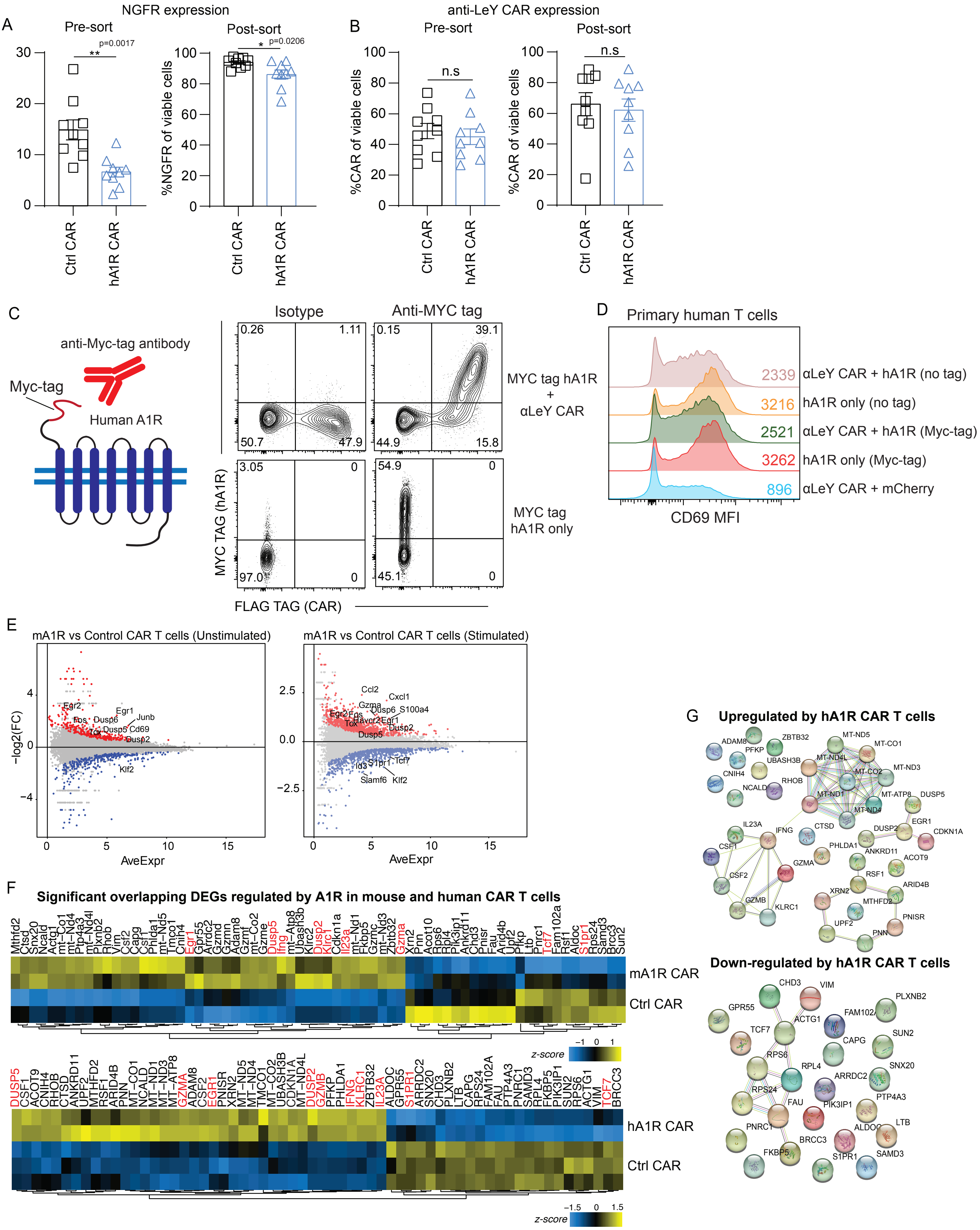
A
NGFR expression
Pre-sort
Post-sort
p=0.0017
**
p=0.0206
*
%NGFR of viable cells
Ctrl CAR
hA1R CAR
B
anti-LeY CAR expression
Pre-sort
Post-sort
n.s
%CAR of viable cells
C
anti-Myc-tag antibody
Myc-tag
Human A1R
Isotype
Anti-MYC tag
MYC tag hA1R + αLeY CAR
MYC tag hA1R only
MYC TAG (hA1R)
FLAG TAG (CAR)
D
Primary human T cells
2339 αLeY CAR + hA1R (no tag)
3216 hA1R only (no tag)
2521 αLeY CAR + hA1R (Myc-tag)
3262 hA1R only (Myc-tag)
896 αLeY CAR + mCherry
CD69 MFI
E
mA1R vs Control CAR T cells (Unstimulated)
mA1R vs Control CAR T cells (Stimulated)
-log2(FC)
AveExpr
F
Significant overlapping DEGs regulated by A1R in mouse and human CAR T cells
mA1R CAR
Ctrl CAR
hA1R CAR
z-score
G
Upregulated by hA1R CAR T cells
Down-regulated by hA1R CAR T cells

Supplementary Figure 2: A_1R transcriptional signature in mouse and human CAR T cells. (A) %NGFR and (B) % anti-Lewis Y CAR expression in pre- and post- NGFR bead sorted human CAR T cells from up to 9 individual experiments. (C) Schematic of MYC-tag human A_1R and anti-myc tag antibody for protein level detection and flow cytometry plots of myc-tag staining of hA_1R versus anti-Lewis Y CAR staining (FLAG). (D) CD69 expression in transduced resting human T cells for hA_1R (myc-tag) versus hA_1R (wild-type). (E) Bulk RNAsequencing of unstimulated or tumor stimulated mouse CAR T cells. (F) Heatmap of overlapping DEGs regulated by both mouse and human A1R CAR T cells versus respective controls. (G) String analysis of protein-protein interactions of overlapping mouse and human A_1R genes in up or down-regulated in human CAR T cells representing the A_1R signature. ****$p<0.0001$, ***$p<0.001$, **$p<0.01$, *$p<0.05$. (a, b) one-way ANOVA. Human bulk RNAseq was performed on a single donor with 3 technical replicates.

Supp Fig 3

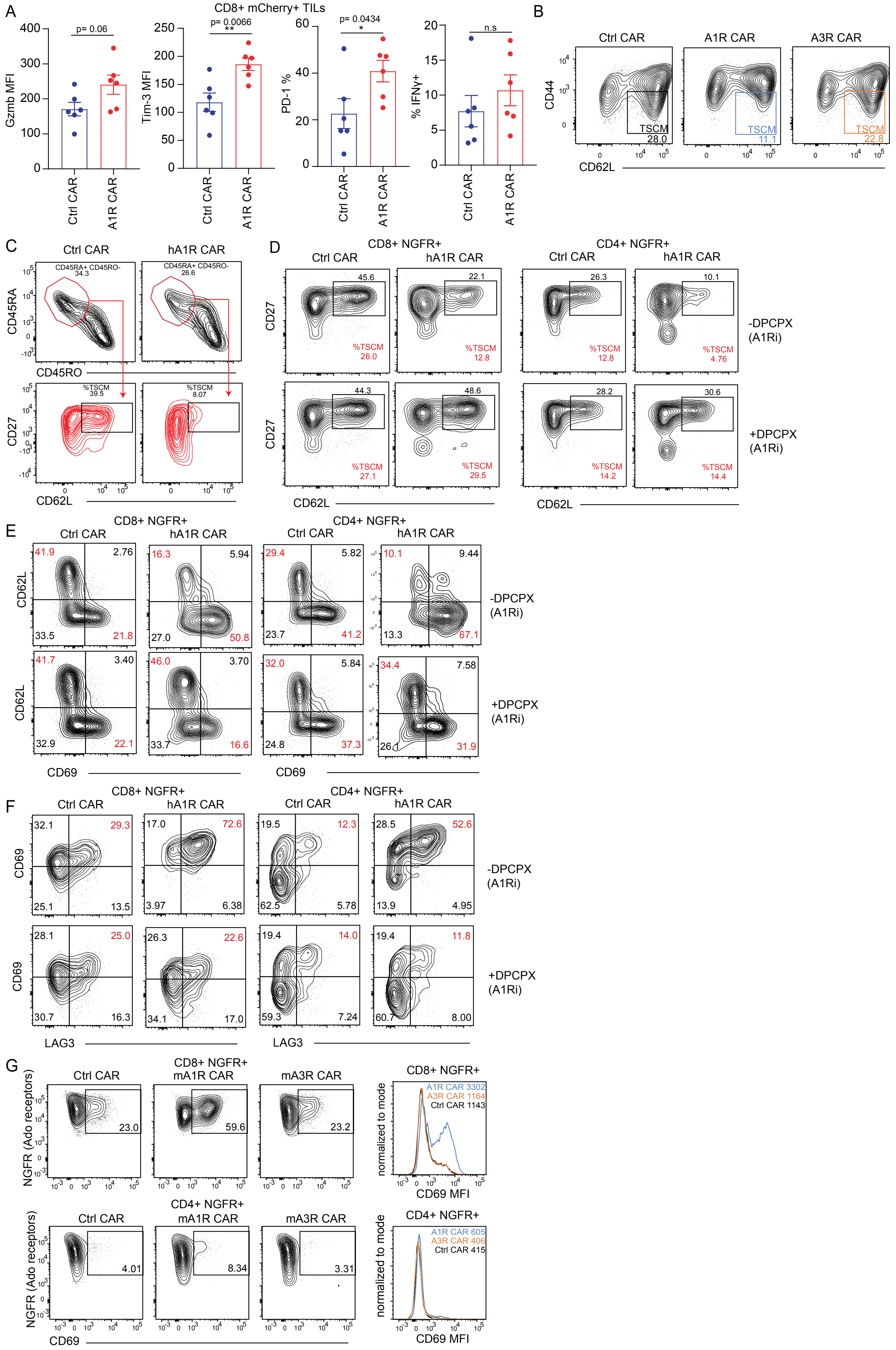
A
CD8+ mCherry+ TILs
p= 0.06
Gzmb MFI
p= 0.0066
**
Tim-3 MFI
p= 0.0434
*
PD-1 %
n.s
% IFNγ+
Ctrl CAR
A1R CAR
B
Ctrl CAR
A1R CAR
A3R CAR
CD44
TSCM
28.0
11.1
22.8
CD62L
C
Ctrl CAR
hA1R CAR
CD45RA+ CD45RO-
34.3
26.6
CD45RA
CD45RO
%TSCM
39.5
8.07
CD27
CD62L
D
CD8+ NGFR+
CD4+ NGFR+
Ctrl CAR
hA1R CAR
45.6
22.1
26.3
10.1
%TSCM
26.0
12.8
12.8
4.76
-DPCPX
(A1Ri)
44.3
48.6
28.2
30.6
27.1
29.5
14.2
14.4
+DPCPX
(A1Ri)
CD27
CD62L
E
CD8+ NGFR+
CD4+ NGFR+
Ctrl CAR
hA1R CAR
CD62L
CD69
41.9
2.76
33.5
21.8
16.3
5.94
27.0
50.8
29.4
5.82
23.7
41.2
10.1
9.44
13.3
67.1
41.7
3.40
32.9
22.1
46.0
3.70
33.7
16.6
32.0
5.84
24.8
37.3
34.4
7.58
26.1
31.9
F
CD8+ NGFR+
CD4+ NGFR+
Ctrl CAR
hA1R CAR
CD69
LAG3
32.1
29.3
25.1
13.5
17.0
72.6
3.97
6.38
19.5
12.3
62.5
5.78
28.5
52.6
13.9
4.95
28.1
25.0
30.7
16.3
26.3
22.6
34.1
17.0
19.4
14.0
59.3
7.24
19.4
11.8
60.7
8.00
G
CD8+ NGFR+
Ctrl CAR
mA1R CAR
mA3R CAR
NGFR (Ado receptors)
23.0
59.6
23.2
normalized to mode
A1R CAR 3302
A3R CAR 1164
Ctrl CAR 1143
CD69 MFI
CD4+ NGFR+
4.01
8.34
3.31
A1R CAR 605
A3R CAR 406
Ctrl CAR 415
CD69

Supplementary Figure 3: A_1R expression drives terminal differentiation and loss of memory precursors *in vitro*. C57BL/6 Her2 transgenic mice were injected orthotopically with 2 x 10^5 E0771-HER2 breast cancer cells in the fourth mammary fat pad. After tumor engraftment, mice were pre-conditioned with 4Gy total body irradiation and treated with 10 x 10^6 Ctrl, A_1R or A_3R mouse anti-HER2 CAR T cells on days 7 and 8 post tumor inoculation. IL-2 (50,000 IU/mouse) was provided I.P on days 0-4 post treatment. (A) Expression of GZMB, TIM3, PD-1 and IFNγ in $CD8^+$ $mCherry^+$ tumor infiltrating CAR T cells was quantified. Data shown as mean ± SEM, from n= 5-6 mice and representative of 2 replicate experiments. (B) Representative FACS plot of TSCM memory subsets of mouse anti-HER2 CAR T cells prior to adoptive transfer. (C) Representative FACS plot of TSCM memory subsets of human anti-Lewis Y CAR T cells prior to adoptive transfer, gated on $CD45RA^+CD45RO^-CD62L^+CD27^+$. (D-F) Representative FACS plots of (D) CD27 versus CD62L gated on $CD45RA^+CD45RO^-$subsets. (E) CD62L versus CD69, (F) CD69versus LAG3 in unstimulated $CD8^+$ or $CD4^+$ human anti-Lewis Y CAR T cells prior to adoptive transfer in the presence or absence of DPCPX (A_1Ri, 100nM). (G) FACS plot and histogram of CD69 expression in unstimulated $CD8^+$/ $CD4^+$ CAR T cells prior to adoptive transfer. ****$p<0.0001$, ***$p<0.001$, **$p<0.01$, *$p<0.05$. (a) one-way ANOVA.

Supp Fig 4

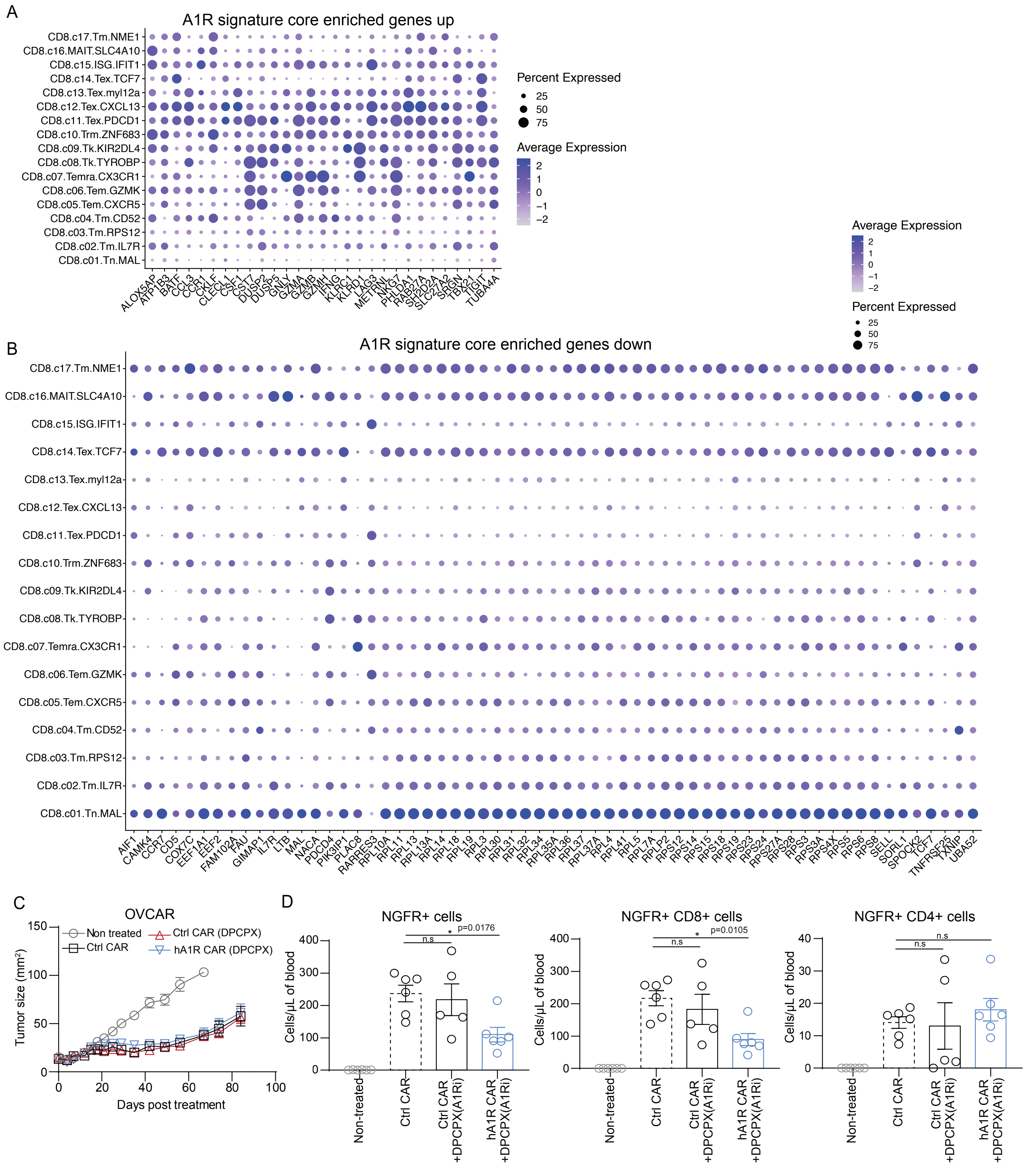

A
A1R signature core enriched genes up
CD8.c17.Tm.NME1
CD8.c16.MAIT.SLC4A10
CD8.c15.ISG.IFIT1
CD8.c14.Tex.TCF7
CD8.c13.Tex.myl12a
CD8.c12.Tex.CXCL13
CD8.c11.Tex.PDCD1
CD8.c10.Trm.ZNF683
CD8.c09.Tk.KIR2DL4
CD8.c08.Tk.TYROBP
CD8.c07.Temra.CX3CR1
CD8.c06.Tem.GZMK
CD8.c05.Tem.CXCR5
CD8.c04.Tm.CD52
CD8.c03.Tm.RPS12
CD8.c02.Tm.IL7R
CD8.c01.Tn.MAL
ALOX5AP ATP1B3 BATF CCL3 CCR1 CKLF CLECL1 CSF1 CST7 DUSP2 DUSP5 GNLY GZMA GZMB GZMH IFNG KLRC1 KLRD1 LAG3 METRNL NKG7 PHLDA1 RAB27A SH2D2A SLC27A2 SRGN TBX21 TIGIT TUBA4A
Percent Expressed
25
50
75
Average Expression
2
1
0
−1
−2
Average Expression
2
1
0
−1
−2
Percent Expressed
25
50
75
B
A1R signature core enriched genes down
CD8.c17.Tm.NME1
CD8.c16.MAIT.SLC4A10
CD8.c15.ISG.IFIT1
CD8.c14.Tex.TCF7
CD8.c13.Tex.myl12a
CD8.c12.Tex.CXCL13
CD8.c11.Tex.PDCD1
CD8.c10.Trm.ZNF683
CD8.c09.Tk.KIR2DL4
CD8.c08.Tk.TYROBP
CD8.c07.Temra.CX3CR1
CD8.c06.Tem.GZMK
CD8.c05.Tem.CXCR5
CD8.c04.Tm.CD52
CD8.c03.Tm.RPS12
CD8.c02.Tm.IL7R
CD8.c01.Tn.MAL
AIF1 CAMK4 CCR7 CD5 COX7C EEF1A1 EEF2 FAM102A FAU GIMAP1 IL7R LTB MAL NACA PDCD4 PIK3IP1 PLAC8 RARRES3 RPL10A RPL11 RPL13 RPL13A RPL14 RPL18 RPL19 RPL3 RPL30 RPL31 RPL32 RPL34 RPL35A RPL36 RPL37 RPL37A RPL4 RPL41 RPL5 RPL7A RPLP2 RPS12 RPS14 RPS15 RPS18 RPS19 RPS23 RPS24 RPS27A RPS28 RPS3 RPS3A RPS4X RPS5 RPS6 RPS8 SELL SORL1 SPOCK2 TCF7 TNFRSF25 TXNIP UBA52
C
OVCAR
Non treated
Ctrl CAR
Ctrl CAR (DPCPX)
hA1R CAR (DPCPX)
Tumor size (mm²)
150
100
50
0
0 20 40 60 80 100
Days post treatment
D
NGFR+ cells
p=0.0176
n.s
*
Cells/μL of blood
400
300
200
100
0
Non-treated
Ctrl CAR
Ctrl CAR +DPCPX(A1Ri)
hA1R CAR +DPCPX(A1Ri)
NGFR+ CD8+ cells
p=0.0105
n.s
*
Cells/μL of blood
400
300
200
100
0
Non-treated
Ctrl CAR
Ctrl CAR +DPCPX(A1Ri)
hA1R CAR +DPCPX(A1Ri)
NGFR+ CD4+ cells
n.s
n.s
Cells/μL of blood
40
30
20
10
0
Non-treated
Ctrl CAR
Ctrl CAR +DPCPX(A1Ri)
hA1R CAR +DPCPX(A1Ri)

Supplementary Figure 4: A_1R signature enriches a transcriptional phenotype positively associated with effector/terminal differentiation and negatively associated with memory T cell subsets. (A-B) Z-score and percentage of gene expression in each meta-cluster GSEA for the full list of core-enriched (A) upregulated or (B) downregulated genes from the A_1R signature as defined by [69]. (C) NSG mice were injected sub-cutaneously with 5 x 10^6 OVCAR-3 tumor cells. Once tumors were established (15-20 mm2), mice were irradiated (1 Gy) and treated with 10 x 10^6 anti-Lewis Y CAR T cells. Where indicated, DPCPX preconditioning was provided to CAR T cells prior to adoptive transfer into mice to preserve memory subsets. Mice were supplemented with 50,000 IU of IL-2 on days 0-4 post treatment. (D) A retro-orbital bleed was performed on day 7 post treatment, and total $NGFR^+$, $CD8^+$ and $CD4^+$ CAR T cells per µL of blood was quantified. (C-D) Data represented as means ± SEM from 1 experiment with n=5-6 mice per group. ****$p<0.0001$, ***$p<0.001$, **$p<0.01$, *$p<0.05$. (c) two-way ANOVA or (d) one-way ANOVA.

Supp Fig 5

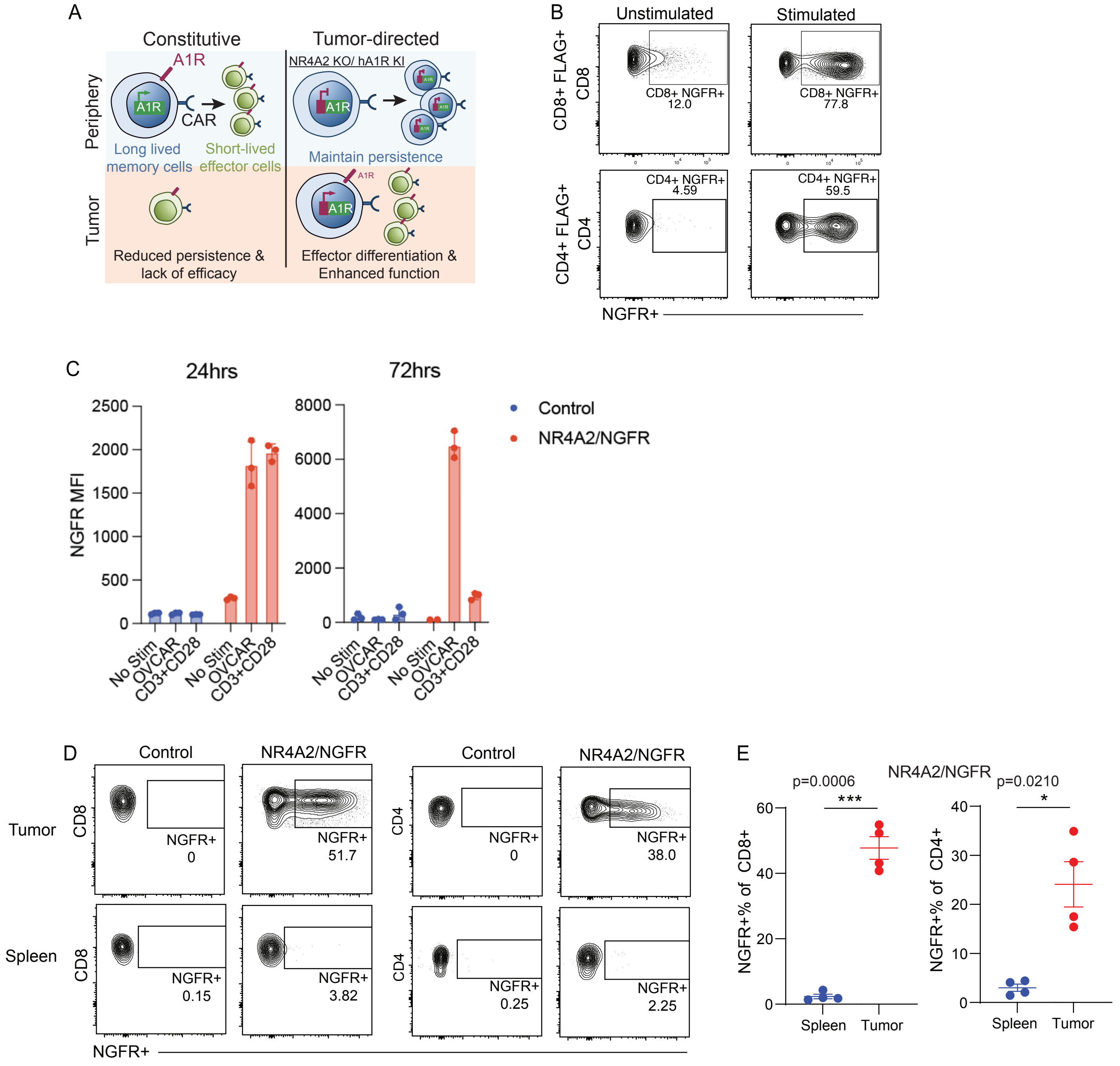
A
Constitutive
Tumor-directed
Periphery
A1R
CAR
Long lived memory cells
Short-lived effector cells
NR4A2 KO/ hA1R KI
Maintain persistence
Tumor
Reduced persistence & lack of efficacy
Effector differentiation & Enhanced function
B
Unstimulated
Stimulated
CD8+ FLAG+
CD8
CD8+ NGFR+ 12.0
CD8+ NGFR+ 77.8
CD4+ FLAG+
CD4
CD4+ NGFR+ 4.59
CD4+ NGFR+ 59.5
NGFR+
C
24hrs
72hrs
NGFR MFI
Control
NR4A2/NGFR
No Stim
OVCAR
CD3+CD28
D
Control
NR4A2/NGFR
Tumor
Spleen
CD8
CD4
NGFR+ 0
NGFR+ 51.7
NGFR+ 38.0
NGFR+ 0.15
NGFR+ 3.82
NGFR+ 0.25
NGFR+ 2.25
NGFR+
E
NR4A2/NGFR
p=0.0006
***
p=0.0210
*
NGFR+% of CD8+
NGFR+% of CD4+
Spleen
Tumor

Supplementary Figure 5: CRISPR knock-in approach for tumor-site directed A_1R expression. (A) Schematic of constitutive versus tumor-directed expression of A_1R in CAR T cells. (B) Flow cytometry plots demonstrating NGFR induction in $CD8^+FLAG^+$ and $CD4^+FLAG^+$ NR4A2/NGFR CAR T cells after 16-hour stimulation with Lewis Y^+ OVCAR-3 tumor cells. (C) Lewis Y-BBz CAR T cells were cocultured with Lewis Y expressing OVCAR- 3 tumor cells or plate-bound αCD3+CD28 for 24 and 72 hours. (D) NSG mice were injected subcutaneously with $5x10^6$ Lewis Y+ OVCAR-3 tumor cells. Once tumors were established (1520 mm2), mice were then irradiated (1 Gy) and treated with one dose of $15x10^6$ anti-Lewis Y CAR T cells. Mice were supplemented with 50,000 IU of IL-2 on days 0-4 post treatment. Flow cytometry analyses of NGFR expression in $CD8^+$ and $CD4^+$ Control and NR4A2/NGFR antiLewis Y CAR T cells in tumor and spleen. (E) Quantification of $NGFR^+$ percentage in $CD8^+$ and $CD4^+$ NR4A2/NGFR CAR T cells in tumors versus spleen. Data represented as means ± SEM of n=4 mice per group from a representative experiment of n = 3. ****p<0.0001, ***p<0.001, **p<0.01, *p<0.05. (d) one-way ANOVA.

Supp Fig 6

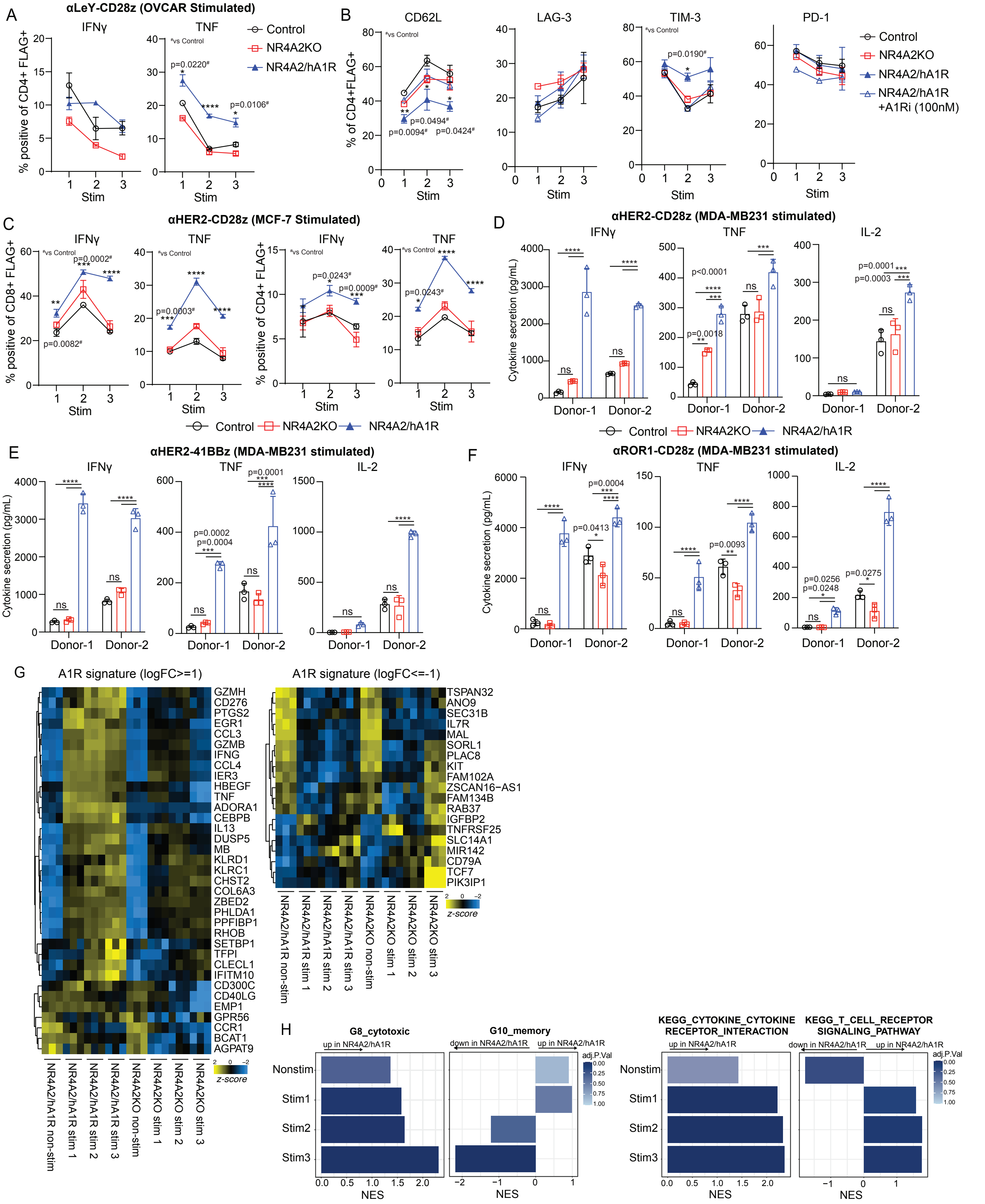
A
αLeY-CD28z (OVCAR Stimulated)
IFNγ
TNF
% positive of CD4+ FLAG+
Stim
#vs Control
p=0.0220#
p=0.0106#
Control
NR4A2KO
NR4A2/hA1R
B
CD62L
LAG-3
TIM-3
PD-1
% of CD4+FLAG+
#vs Control
p=0.0494#
p=0.0094#
p=0.0424#
p=0.0190#
Stim
Control
NR4A2KO
NR4A2/hA1R
NR4A2/hA1R
+A1Ri (100nM)
C
αHER2-CD28z (MCF-7 Stimulated)
IFNγ
TNF
% positive of CD8+ FLAG+
% positive of CD4+ FLAG+
#vs Control
p=0.0002#
p=0.0082#
p=0.0003#
p=0.0243#
p=0.0009#
Stim
Control
NR4A2KO
NR4A2/hA1R
D
αHER2-CD28z (MDA-MB231 stimulated)
IFNγ
TNF
IL-2
Cytokine secretion (pg/mL)
p<0.0001
p=0.0018
p=0.0001
p=0.0003
ns
Donor-1
Donor-2
Control
NR4A2KO
NR4A2/hA1R
E
αHER2-41BBz (MDA-MB231 stimulated)
IFNγ
TNF
IL-2
Cytokine secretion (pg/mL)
p=0.0001
p=0.0002
p=0.0004
ns
Donor-1
Donor-2
F
αROR1-CD28z (MDA-MB231 stimulated)
IFNγ
TNF
IL-2
Cytokine secretion (pg/mL)
p=0.0004
p=0.0413
p=0.0093
p=0.0256
p=0.0248
p=0.0275
ns
Donor-1
Donor-2
G
A1R signature (logFC>=1)
GZMH
CD276
PTGS2
EGR1
CCL3
GZMB
IFNG
CCL4
IER3
HBEGF
TNF
ADORA1
CEBPB
IL13
DUSP5
MB
KLRD1
KLRC1
CHST2
COL6A3
ZBED2
PHLDA1
PPFIBP1
RHOB
SETBP1
TFPI
CLECL1
IFITM10
CD300C
CD40LG
EMP1
GPR56
CCR1
BCAT1
AGPAT9
A1R signature (logFC<=-1)
TSPAN32
ANO9
SEC31B
IL7R
MAL
SORL1
PLAC8
KIT
FAM102A
ZSCAN16-AS1
FAM134B
RAB37
IGFBP2
TNFRSF25
SLC14A1
MIR142
CD79A
TCF7
PIK3IP1
z-score
NR4A2/hA1R non-stim
NR4A2/hA1R stim 1
NR4A2/hA1R stim 2
NR4A2/hA1R stim 3
NR4A2KO non-stim
NR4A2KO stim 1
NR4A2KO stim 2
NR4A2KO stim 3
H
G8_cytotoxic
up in NR4A2/hA1R
G10_memory
down in NR4A2/hA1R
up in NR4A2/hA1R
KEGG_CYTOKINE_CYTOKINE
RECEPTOR_INTERACTION
KEGG_T_CELL_RECEPTOR
SIGNALING_PATHWAY
adj.P.Val
Nonstim
Stim1
Stim2
Stim3
NES

Supplementary Figure 6: Tumor-site directed A_1R expression enhanced cytokine production in CAR T cells targeting Lewis Y, HER2 and ROR1 antigens and drives A_1R signature and phenotype upon tumor stimulation. (A) Intracellular staining of cytokines in $CD4^+FLAG^+$ CAR T cells after OVCAR-3 serial tumor stimulation. (B) Flow cytometry analysis of $CD4^+FLAG^+$ anti-Lewis Y-CD28z CAR T cell markers in OVCAR-3 tumor serial stimulation assay in the presence or absence of DPCPX (100nM). (C) Intracellular staining of cytokines in $CD8^+$ and $CD4^+$ anti-HER2-CD28z CAR T cells after coculture with MCF-7 $HER2^+$ tumors. (D) Cytokine secretion by anti-HER2-CD28z and (E) 41BBz CAR T cells after serial tumor stimulation for 72 hours with $HER2_{low}$ MDA-MB231 breast cancer cells. (F) Cytokine secretion by anti-ROR1-CD28z CAR T cells against $ROR1^+$ MDA-MB231 tumors. (A-F) Data represented as means ± SD of triplicate cultures from a representative experiment of n = 3. (G) Heatmap of A_1R gene signature derived from constitutive overexpression of A_1R in mouse and human CAR T cells, filtered on log fold change of greater than 1 or less than -1. (H) GSEA barplots of Sade Feldman (GSE120575) and KEGG pathways in anti-Lewis Y NR4A2/hA_1R vs Control CAR T cells after OVCAR-3 serial tumor stimulation. ****$p<0.0001$, ***$p<0.001$, **$p<0.01$, *$p<0.05$. (A-F) two-way ANOVA. (D-F) Representative data for n=2 healthy donors.

Supp Fig 7

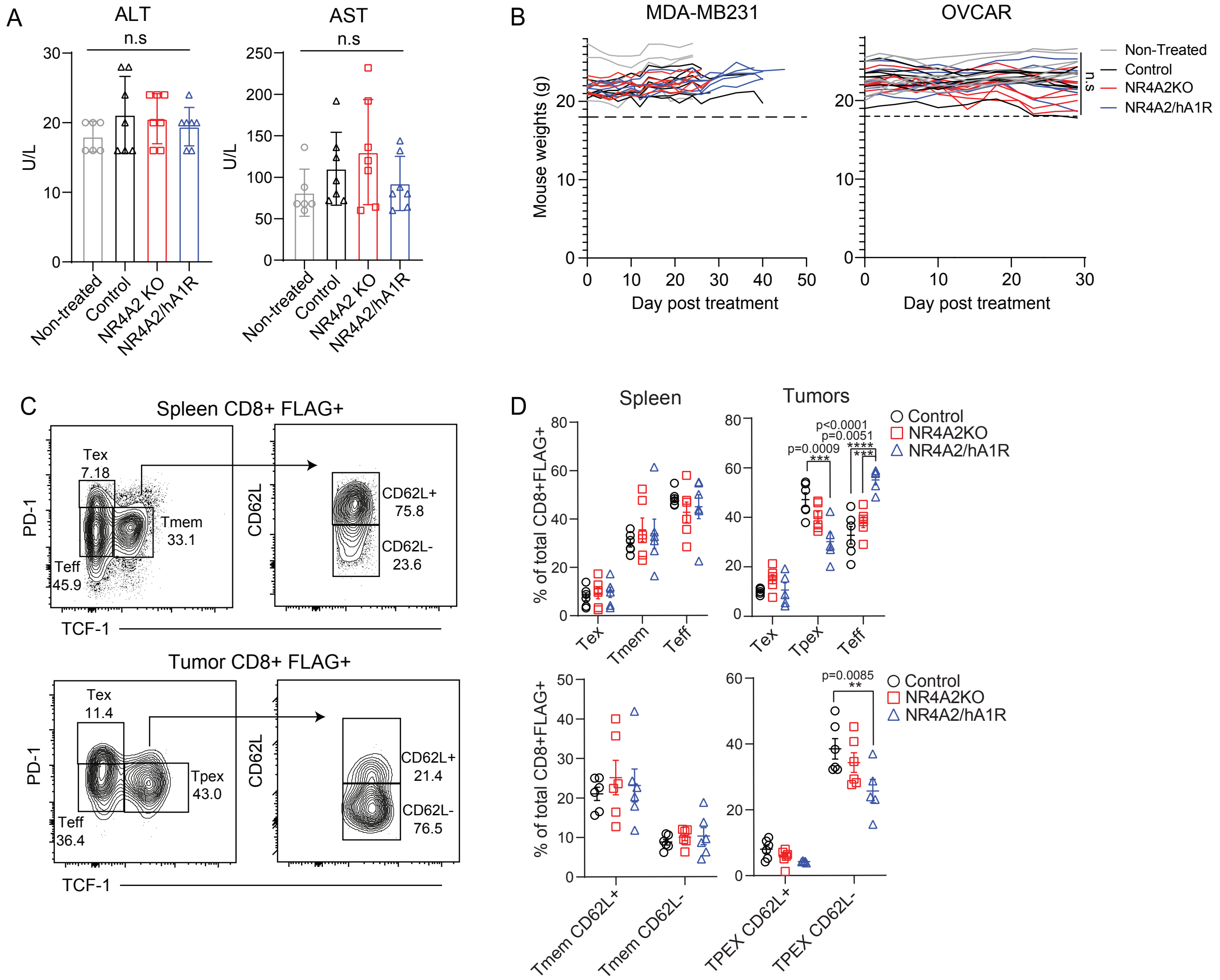
A
ALT
n.s
U/L
AST
n.s
Non-treated
Control
NR4A2 KO
NR4A2/hA1R
B
MDA-MB231
OVCAR
Mouse weights (g)
Day post treatment
n.s
Non-Treated
Control
NR4A2KO
NR4A2/hA1R
C
Spleen CD8+ FLAG+
Tex 7.18
Tmem 33.1
Teff 45.9
PD-1
TCF-1
CD62L
CD62L+ 75.8
CD62L- 23.6
Tumor CD8+ FLAG+
Tex 11.4
Tpex 43.0
Teff 36.4
CD62L+ 21.4
CD62L- 76.5
D
Spleen
Tumors
% of total CD8+FLAG+
Tex
Tmem
Teff
Tpex
p<0.0001
p=0.0051
p=0.0009
Control
NR4A2KO
NR4A2/hA1R
p=0.0085
Tmem CD62L+
Tmem CD62L-
TPEX CD62L+
TPEX CD62L-

Supplementary Figure 7: Tumor site directed A_1R expression drives CAR T cells effector differentiation within tumors and not spleen *in vivo*. NSG mice were injected subcutaneously with 5 x 10^6 OVCAR-3 tumor cells. Once tumors were established (15-20 mm^2), mice were then irradiated (1 Gy) and treated with one dose of $15x10^6$ anti-Lewis Y CAR T cells. Mice were supplemented with 50,000 IU of IL-2 on days 0-4 post treatment. (A) Concentration of enzymes associated with liver toxicity from serum collected from OVCAR-3 tumor bearing mice treated with CAR T cells. Data represented as means ± SEM from 1 experiment with n=6-7 mice per group. (B) Mouse weights over duration therapy. (C) Gating strategy for tumor infiltrating $CD62L^{+/-}$ T_{PEX}, splenic $CD62L^{+/-}$ T_{MEM}, T_{EX} and T_{EFF} memory subsets across spleen and tumor infiltrating $CD8^+FLAG^+$ CAR T cells. (D) Quantification of memory subsets percentages. Data represented as means ± SEM from 1 experiment with n=56 mice per group. ****p<0.0001, ***p<0.001, **p<0.01, *p<0.05. (b,d) two-way ANOVA or (a) one-way ANOVA.

Supp Fig 8

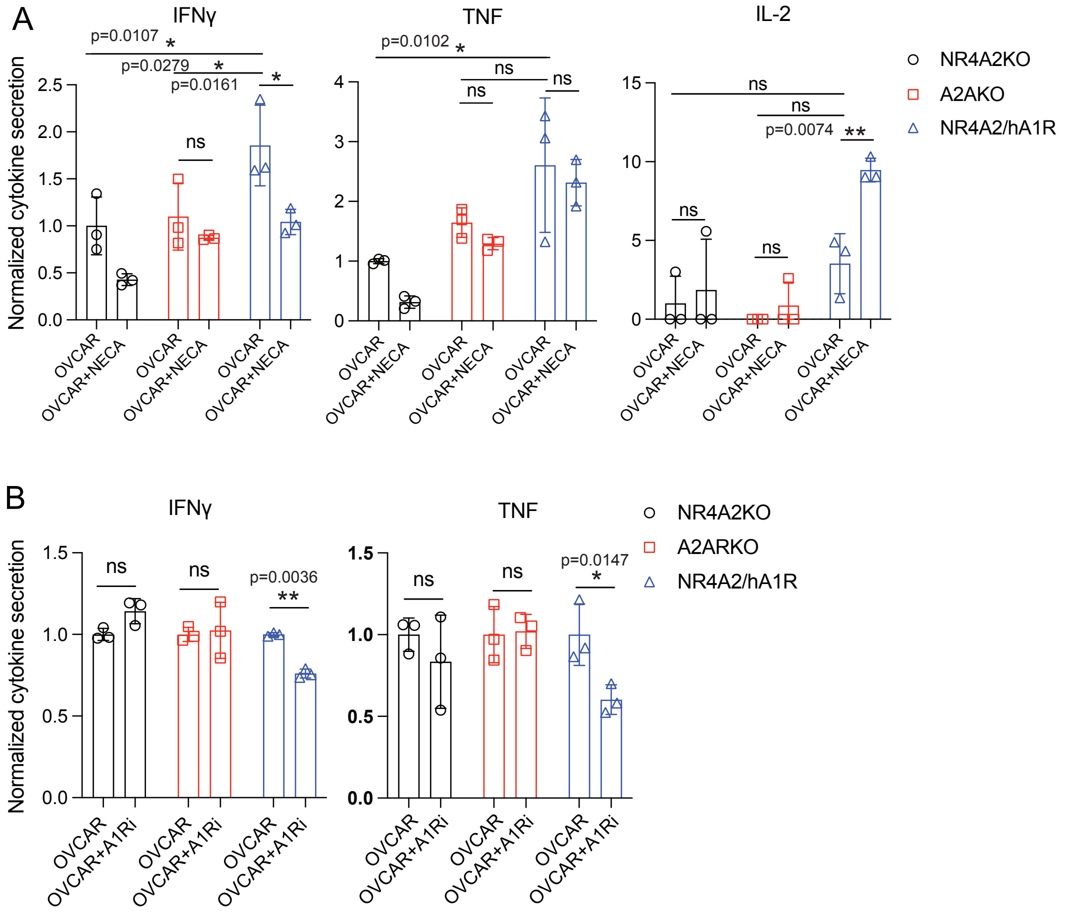
A
IFNγ
TNF
IL-2
Normalized cytokine secretion
p=0.0107
p=0.0279
p=0.0161
p=0.0102
p=0.0074
ns
NR4A2KO
A2AKO
NR4A2/hA1R
OVCAR
OVCAR+NECA
B
IFNγ
TNF
p=0.0036
p=0.0147
NR4A2KO
A2ARKO
NR4A2/hA1R
OVCAR
OVCAR+A1Ri

Supplementary Figure 8: A_1R expression and $A_{2A}R$ deletion in CAR T cells differentially regulate cytokine expression. Cytokine production in CAR T cells that were serially stimulated with OVCAR-3 tumors for 72 hours in the presence or absence of (A) pan-adenosine receptor agonist NECA (10μM) normalized against NR4A2 CAR-T cells without NECA or (B) A_1R antagonist DPCPX (1μM) normalized within each group without DPCPX. ****p<0.0001, ***p<0.001, **p<0.01, *p<0.05. (a, b) two-way ANOVA. Data represented as mean ± SD of triplicate cultures from 2 independent donors.

Supp Fig 9

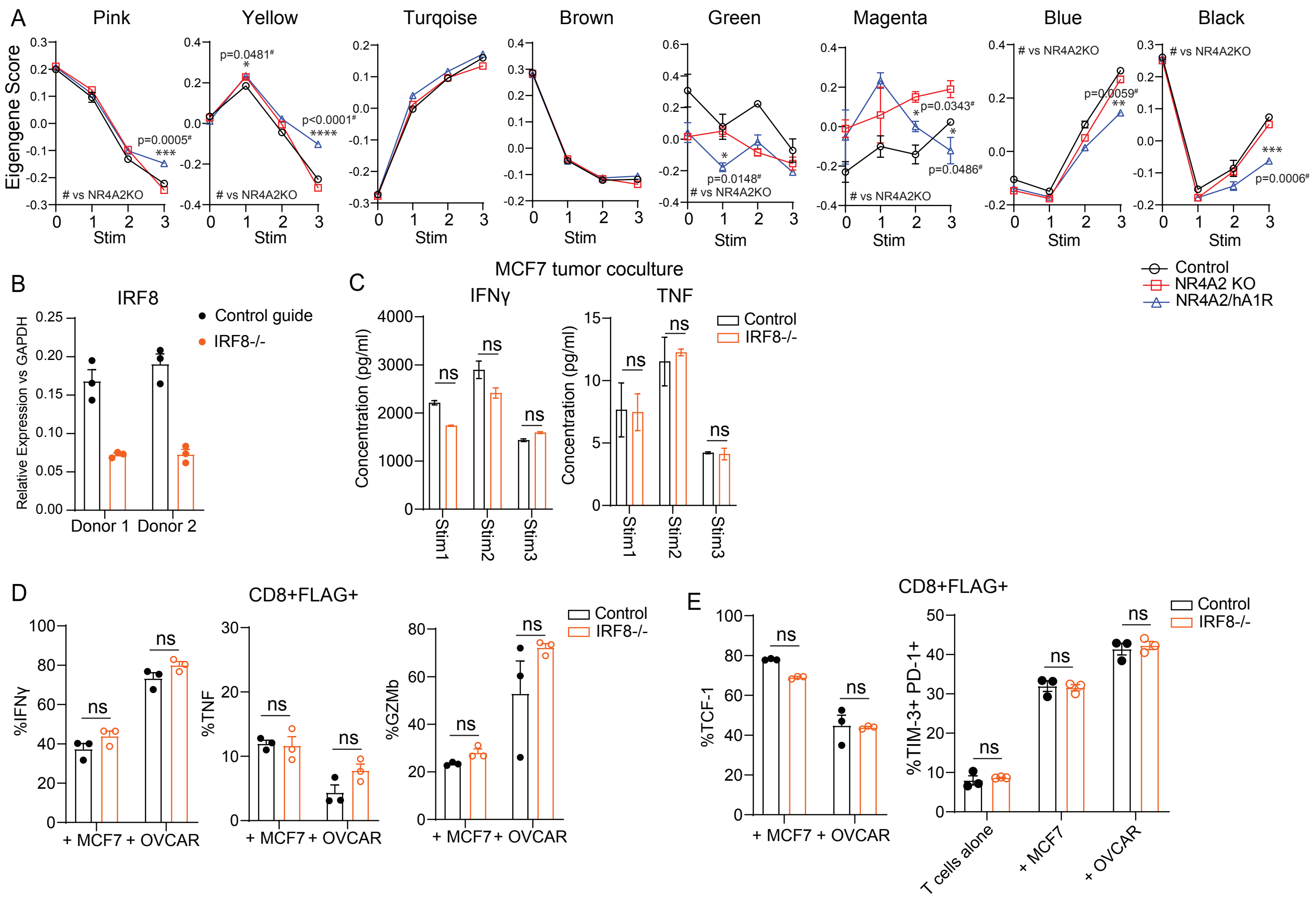
A
Pink
Yellow
Turqoise
Brown
Green
Magenta
Blue
Black
Eigengene Score
Stim
# vs NR4A2KO
p=0.0005#
***
p=0.0481#
*
p<0.0001#
****
p=0.0148#
p=0.0343#
p=0.0486#
p=0.0059#
**
p=0.0006#
Control
NR4A2 KO
NR4A2/hA1R
B
IRF8
Control guide
IRF8-/-
Relative Expression vs GAPDH
Donor 1
Donor 2
C
MCF7 tumor coculture
IFNγ
TNF
Concentration (pg/ml)
Control
IRF8-/-
ns
Stim1
Stim2
Stim3
D
CD8+FLAG+
%IFNγ
%TNF
%GZMb
+ MCF7
+ OVCAR
E
CD8+FLAG+
%TCF-1
%TIM-3+ PD-1+
T cells alone
+ MCF7
+ OVCAR

Supplementary Figure 9: IRF8 deletion does not affect control CAR T cell phenotype or cytokine production. (A) Eigengene score in each module identified by WGCNA analyses at each round of stimulation. Significance calculated using a two-way ANOVA and shown for NR4A2/hA$_1$R vs NR4A2 control. (B) Validation of IRF8 guides based on qRT-PCR expression in human anti-Lewis Y CAR T cells. Data represented as means ± SD of triplicate samples. (C) Cytokine secretion and (D) intracellular staining of cytokines and granzyme B gated on CD8$^+$ FLAG$^+$ IRF8KO and control CAR T cells. (E) Memory and exhaustion markers on IRF8KO CAR T cells stimulated with MCF-7 tumors after 3 rounds of serial stimulation. (D-E) Data represented as means ± SD of triplicate cultures from a representative experiment of n = 2. ****p<0.0001, ***p<0.001, **p<0.01, *p<0.05. (a) two-way ANOVA or (c, d, e) one-way ANOVA.

Supp Fig 10.

A

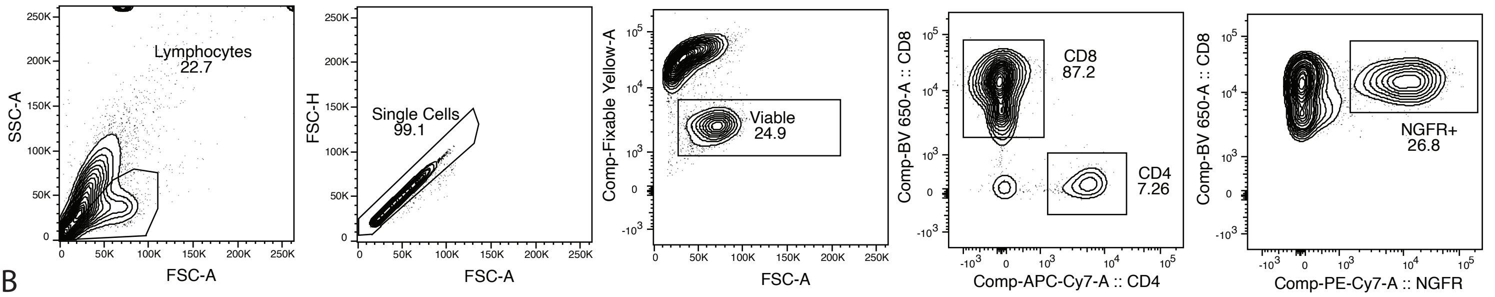

B

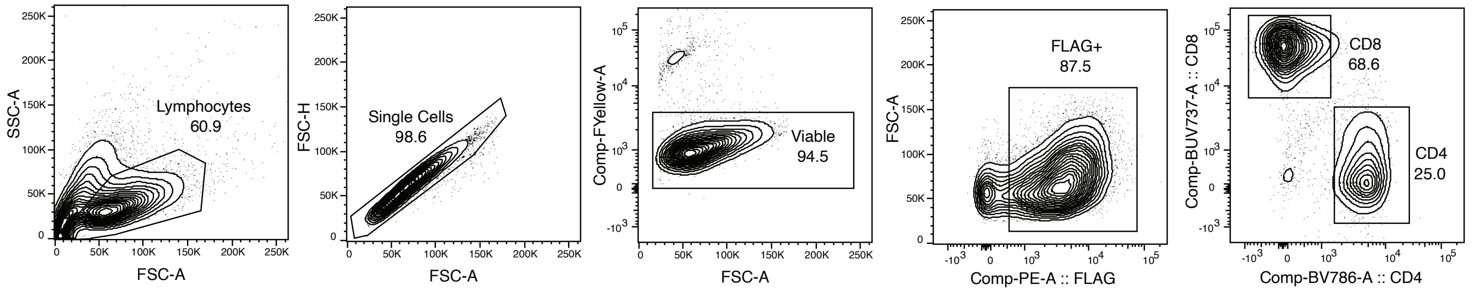

Supplementary Figure 10: Gating Strategy for the analysis of mouse and human CAR T cells. (A) Mouse CAR T cells. (B) Human CAR T cells. FSC/SSC gate was first used to gate on the morphology of leukocytes. Cell doublets were excluded by gating on the diagonal of FSC-A vs FSC-H followed by a viability gate (LIVE/DEAD$_{TM}$ Fixable Yellow (thermofisher) or DAPI) to excluded dead cells. CAR T cells were identified using a T cell lineage marker (Thy1.2 or CD3), CD8 or CD4 lineage markers and transduction markers (mCherry, NGFR or FLAG).
