# Supplementary material for: Tumor site-directed A1R expression enhances CAR T cell function and improves efficacy against solid tumors
Source: Nat Commun. 2025 Jul 3;16:6123. doi: 10.1038/s41467-025-59021-9 (PMC12229354; doi:10.1038/s41467-025-59021-9)
Supplement: Supplementary file 2 — Description of Additional Supplementary Files [file 41467_2025_59021_MOESM2_ESM.pdf]

Description of Additional Supplementary Files

Supplementary Data 1: MYC-tagged hA1R peptide sequence
